# Improved photorespiration has a major impact on the root metabolome of Arabidopsis

**DOI:** 10.1111/ppl.70142

**Published:** 2025-03-03

**Authors:** Stefan Timm, Alexandra Florian, Saleh Alseekh, Kathrin Jahnke, Martin Hagemann, Alisdair R. Fernie, Hermann Bauwe

**Affiliations:** ^1^ Plant Physiology Department University of Rostock Rostock Germany; ^2^ Max Planck Institute of Molecular Plant Physiology Germany

## Abstract

Photorespiration is an essential metabolic repair process in oxygenic photosynthesis, as it detoxifies Rubisco's inhibitory oxygenase byproduct, 2‐phosphoglycolate (2‐PG). It has been demonstrated that improving endogenous photorespiration in C3 plants through enzyme overexpression can enhance photosynthesis and promote plant growth. However, the potential impact of improved photorespiration in leaves on heterotrophic roots remained unexplored. To address this, we conducted a metabolome analysis of Arabidopsis leaves and roots using transgenic lines with enhanced glycine decarboxylase (GDC) activity, achieved by overexpressing the mitochondrial lipoamide dehydrogenase (mtLPD1) subunit. In the leaves, *mtLPD1* overexpression primarily resulted in reduced steady‐state levels of intermediates associated with photorespiration, the tricarboxylic acid (TCA) cycle, and soluble sugars, while intermediates related to nitrogen metabolism were elevated. In roots, where *mtLPD1* expression was unchanged, we observed contrasting accumulation patterns in the transgenic lines compared to the wildtype, including increased levels of photorespiratory and TCA‐cycle intermediates. Notably, we also detected elevated amounts of soluble sugars, nitrate, and starch. Phloem exudate analysis revealed altered metabolite profiles in the overexpressors, particularly with respect to photorespiratory intermediates linked to the GDC reaction, as well as soluble sugars and metabolites involved in cellular redox homeostasis. This suggested an increased transport of these metabolites from shoots to roots, thereby altering sink organ metabolism. In summary, we hypothesize that optimizing photorespiration enhances photosynthesis, which leads to an increased export of carbon surplus to heterotrophic tissues. Thus, improving photorespiration may hold potential for increasing yields in beet‐ and tuber‐forming plants.

## 1 INTRODUCTION

Photorespiration is indispensable for oxygenic phototrophs in today's high oxygen‐containing atmosphere. The main foundation for this statement lies in the biochemical properties of the world's predominant CO_2_‐fixing enzyme, ribulose‐1,5‐bisphosphate (RuBP) carboxylase/oxygenase (Rubisco), as it is incapable of fully discriminating between CO_2_ and O_2_. Whilst the carboxylation reaction of RuBP produces two 3‐phosphoglycerate (3‐PGA) molecules that enter the Calvin‐Benson (CB) cycle for biosynthesis of complex carbohydrates, oxygenation of RuBP yields only one 3‐PGA alongside with the production of one 2‐phosphoglycolate (2‐PG) (Bauwe et al., 2012; Busch et al., 2020). 2‐PG accumulation is critical for oxygenic phototrophs since it cannot directly enter the CB‐cycle for further metabolization in conjunction with the inhibition of a few key carbon‐utilizing enzymes such as phosphofructokinase (PFK), sedoheptulose‐1,7‐bisphosphatase (SBPase) and triosephosphate phosphatase (TPI) (Kelly and Latzko, 1976; Flügel et al., 2017; Li et al., 2019). For this reason, photorespiration coevolved to oxygenic photosynthesis to operate as its primary metabolic repair shunt, essentially reconverting two 2‐PG molecules back into one 3‐PGA, thereby releasing CO_2_ and ammonia (Eisenhut et al., 2008; Bauwe et al., 2012).

Over the past two decades, significant progress has been made in studying and engineering plant photorespiration, especially aiming at the identification of strategies to increase plant yield (e.g. Peterhänsel et al., 2013; Betti et al., 2016; South et al., 2018). This is because, although photorespiration is essential to maintain optimal photosynthesis in the presence of oxygen, the pathway itself operates with a considerable yield penalty. Pending on environmental conditions, this manifests in a net loss of up to 30% of previously fixed carbon as CO_2_ to the atmosphere during glycine cleavage by glycine decarboxylase (GDC), accompanied by marked energetic costs (Walker et al., 2016; South et al., 2018). Therefore, most of the research efforts are based on circumventing the GDC reaction through the introduction of artificial bypasses that release CO_2_ in the chloroplasts in order to decrease the oxygenase activity of Rubisco and potentially lower the amount of ammonia simultaneously released by the same reaction (Peterhänsel et al., 2013; South et al., 2018; Eisenhut et al., 2019). Interestingly, some benefits were observed in the bypass plants in different plant species under laboratory and field conditions, including future climates (Kebeish et al., 2007; South et al., 2019; Meacham‐Hensold et al., 2024; Sun et al., 2024). Another successful strategy aims to improve endogenous photorespiration through overexpression of specific pathways enzymes. More precisely, overexpression of 2‐PG phosphatase (PGLP; Flügel et al., 2017) or GDC subunits H (Timm et al., 2012a) or L protein (Timm et al., 2015) lowered the steady‐state contents of certain photorespiratory intermediates. In conjunction with ^13^C‐labelling experiments, these findings suggested a faster flux through the improved natural photorespiratory cycle (Timm et al., 2015). This metabolic chassis is hypothesized to build the basis for reduced negative feedback inhibition on carbon assimilation and downstream utilization (Fernie and Bauwe, 2020; Timm and Hagemann, 2020). Similar to the observations with some of the bypass plants, stimulation of photosynthesis and growth through photorespiratory optimization was seen under both laboratory and field conditions in different plant species (Timm et al., 2012a, 2015; Simkin et al., 2017; López‐Calcagno et al., 2019). Collectively, it seems reasonable to conclude that, irrespective of the strategy, beneficial effects on photosynthesis and growth are a direct consequence of lower abundances of specific photorespiratory intermediates.

Given that photorespiration is restricted to illuminated, photosynthesizing tissues, its impact on other non‐photosynthesizing plant tissues or during darkness is yet understudied. This correlates well with the absence or inactivity of Rubisco in these organs and in the absence of light, respectively (Ogren et al., 1986). Lower abundances of photorespiratory intermediates in the pathway mutants during the night support this statement (Timm et al., 2012b, 2013). Furthermore, an initial study provided evidence for the presence of key photorespiratory intermediates in heterotrophic tissues and several pathway enzymes were found to be also expressed in roots (Nunes‐Nesi et al., 2014). Hence, some photorespiratory enzymes might be active in the dark and in heterotrophic tissues in order to drive downstream processing of critical intermediates. Based on these findings and the yet unresolved question of what is the main sink of the surplus of photosynthetically fixed carbon in the transgenic *Arabidopsis thaliana* (Arabidopsis) lines with increased photorespiratory flux, we conducted an organ‐specific metabolomic analysis. Here, we compared metabolite accumulation patterns of Arabidopsis wildtype and GDC L‐protein (mtLPD1) overexpression plants (Timm et al., 2015) during three experimental setups: (1) plants were grown on soil and sampled during illumination and darkness to quantify photorespiratory intermediates with active and inactive photorespiration, (2) plants were grown hydroponically to analyze for changes in leaves and roots of the same plant in response to altered photorespiratory fluxes, and finally (3) plants were used to analyze the phloem metabolite composition to gain insight into potential metabolite‐driven communication between the source (leaves) and sink (roots) tissue. Collectively, we found comparable metabolite accumulation patterns between soil and hydroponic growth. Further, our results revealed a severe impact of altered leaf‐photorespiratory fluxes on heterotrophic tissues beyond that of photorespiration. In the phloem, we observed specific metabolite changes, essentially involving the photorespiratory metabolite glycine, soluble sugars, and redox‐related compounds. The results suggest their potential role as transport or signaling metabolite, communicating altered photosynthetic‐photorespiratory capacities onto storage organs to adapt their metabolism. The obtained results are discussed in terms of their potential to improve genetic engineering strategies for increased yields of beet‐ or tuber‐forming plants such as sugar beet, potato or cassava.

## 2 MATERIAL AND METHODS

### 2.1 Plant material and growth

**FIGURE 1:**
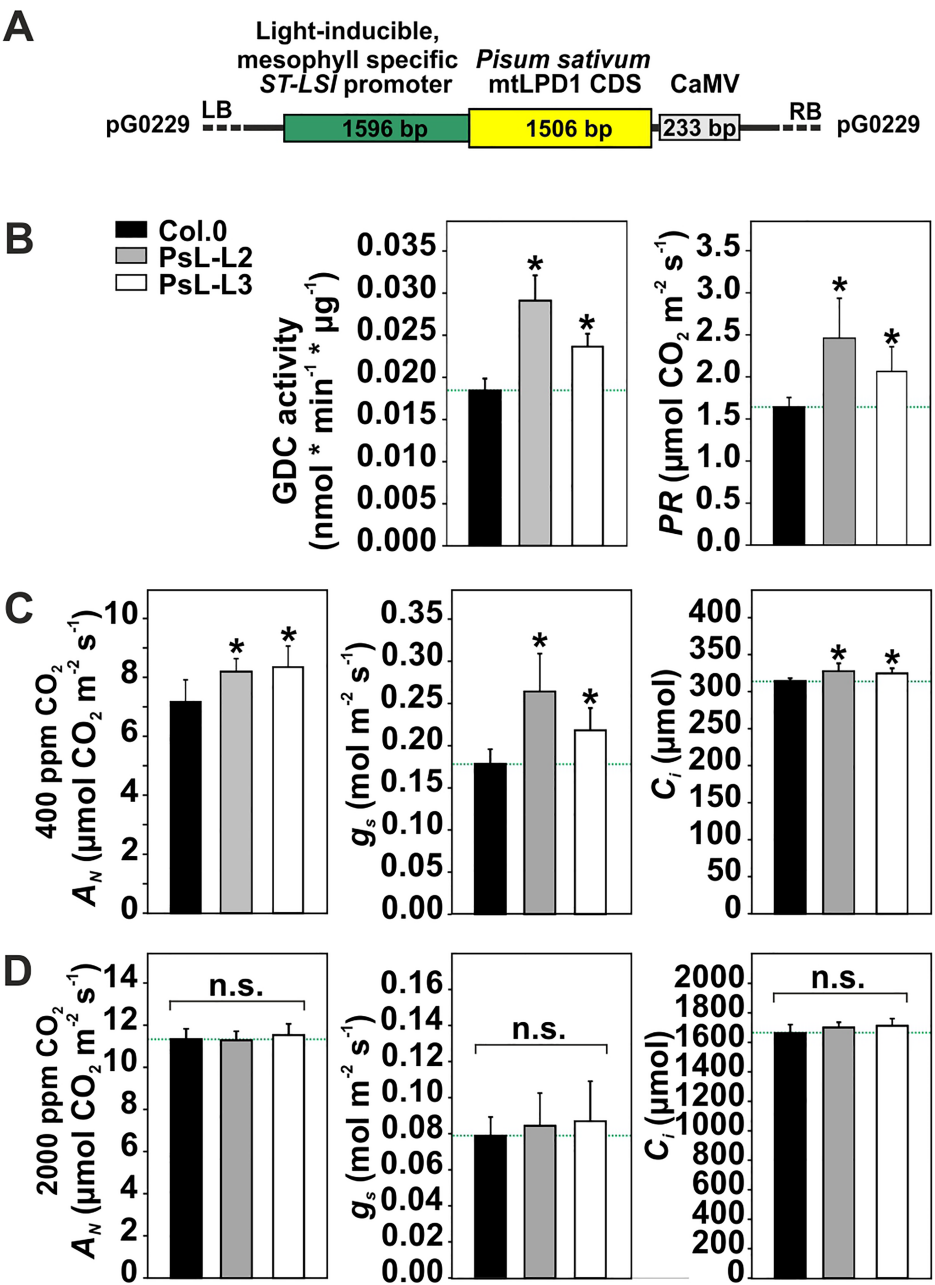
Construct, GDC activity and selected photosynthetic‐photorespiratory gas exchange parameters of *mtLPD1* overexpressors and the wildtype. (A) Scheme of the *mtLPD1* overexpression construct. (B) GDC activity in isolated mitochondria and rate of photorespiration. Selected gas exchange parameters of soil‐grown plants at growth stage 5.1 (Boyes et al., 2001) measured with (C) 400 ppm CO_2_ and a light intensity of 200 μmol photos m^−2^ s^−1^ and (D) 2000 ppm CO_2_ and a light intensity of 200 μmol photos m^−2^ s^−1^. Values are means of at least 4 biological replicates. Asterisks determine values significantly different from the wildtype control based on Student's t‐test (*p < 0.05).

During this study, *Arabidopsis thaliana* (Arabidopsis) ecotype Columbia 0 (Col.0) was used as wildtype reference alongside two previously characterized glycine decarboxylase (GDC) L‐protein (mtLPD1; EC:1.8.1.4.) overexpression lines (designated as PsL‐L2 and PsL‐L3) with optimized photorespiration (Timm et al., 2015). To prevent from major artificial side effects on other metabolisms, overexpression is driven by the light‐inducible, mesophyll‐specific *ST‐LSI* promoter (Stockhaus et al., 1989; Figure 1A) to regulate the expression of the mtLPD1 surplus similar to other photosynthetic and photorespiratory genes. Prior to sowing on half‐strength Murashige and Skoog (MS) media including vitamins and 0.5% sucrose, seeds were surface sterilized through hypochloric acid treatment and incubated for 2 days at 4°C to break dormancy. Following germination, plants were transplanted and grown on soil (4:1 mixture of soil [Type Mini Tray; Einheitserdewerk; Percival growth cabinets] and vermiculite and regularly watered with 0.2% Wuxal liquid fertilizer [Aglukon]). For some experiments, plants were cultivated hydroponically as described earlier (Nunes‐Nesi et al., 2014) in controlled environmental chambers (SANYO). During hydroponic growth, roots were bubbled with normal air (21% O_2_; 0.040% CO_2_) and fully covered from light to allow for comparisons with soil‐grown plants. All experiments were performed with the following standard growth conditions: 10/14‐h day/night cycle, 20/18°C day/night temperature, ~120 μmol m^−2^ s^−1^ irradiance, 400 mL L^−1^ CO_2_, and ~ 70% relative humidity. For all analyses, we used plants at growth stage 5.1 (Boyes et al., 2001).

### 2.2 Gas exchange measurements

In order to determine photosynthetic‐photorespiratory parameters, we performed standard leaf gas exchange measurements on fully expanded rosette leaves of plants at growth stage 5.1 (Boyes et al., 2001) with the following conditions: photon flux density = 200 μmol m^−2^ s^−1^, chamber temperature = 25°C, flow rate = 300 μmol s^−1^, and a relative humidity of ~70%. A/C_i_ curves were recorded after at least 10 minutes of adaptation to the measuring conditions. Photosynthetic net CO_2_ assimilation (*A*_*N*_), stomatal conductance (*g*_*s*_) and the intracellular CO_2_ concentration (*C*_*i*_) were determined at air CO_2_ levels (400 ppm) and in elevated CO_2_ (2000 ppm), suppressing photorespiration. Rates of photorespiration were calculated as described previously (Timm et al., 2015), following the model proposed by Sharkey (1988).

### 2.3 Isolation of Mitochondria and GDC activity

Mitochondria were isolated from leaves of wildtype and transgenic plants grown in normal air to stage 5.1 (Boyes et al., 2005) according to Keech et al. (2005). GDC activity was determined as described previously (Hasse et al., 2007) with some modifications. Briefly, mitochondrial proteins were extracted and rebuffered in 20 mM Na‐phosphate (pH 6.0), following 3 sonification cycles of 15‐sec each and centrifugation (20000 x *g*, for 15 min at 4°C). Protein concentrations were estimated according to Bradford (1976). GDC activity was assayed as P‐protein activity by measuring the exchange of the carboxyl carbon of glycine against ^14^C‐bicarbonate carbon (Klein and Sagers, 1966). The standard assay mixture, in a total volume of 450 μL at 30°C, contained 20 mM Na‐phosphate (pH 6.0), 0.1 mM pyridoxal phosphate, 1 mM dithiothreitol, 20 mM glycine, 30 mM NaH^14^CO_3_, 20 μg mitochondrial protein. All rates were corrected by control reactions without glycine. Subsequent to a 3 min pre‐incubation, reactions were started by the addition of NaH^14^CO_3_. After 0, 20, 40 and 60 min, 100 μL reaction mixture were mixed with 50 μL trichloroacetic acid and dried overnight on a hot plate to remove all ^14^C‐bicarbonate. Residues were dissolved in 450 μL sterile water, and the ^14^C‐glycine was quantified by liquid scintillation counting.

### 2.4 Metabolite analysis by GC–MS

For gas chromatography coupled to mass spectrometry (GC–MS) analysis, we harvested leaf and root materials from the same plant at the midday (MoD; 5 h light) of hydroponically‐grown plants or at MoD and end of the night (EoN; 13 h darkness) of soil‐gown plants. The plant tissue was immediately frozen in liquid nitrogen and stored at −80°C until analysis. Approximately 30–50 mg of fully expanded rosette leaves or roots from at least five biological replicates were used for GC–MS analysis. Metabolite extraction, derivatization, and analysis were performed exactly as described by Lisec et al. (2006). For absolute metabolite quantification of photorespiratory intermediates, authentic standard substances were used at varying concentrations for calibration and normalization. To analyze the phloem metabolite composition, soil grown plants were used at MoD. The exudates were collected during a 1‐hour incubation phase in the dark, essentially following the procedure described earlier (Tetyuk et al., 2013). Five phloem exudates as pools of 3–4 different leaves harvested from 3 individual biological replicates were analyzed per genotype according to the well‐established standard method (Lisec et al., 2006).

### 2.5 Quantification of starch, nitrate and ammonium

Starch, ammonium and nitrate contents were quantified from ~30–50 mg of leaf and root tissues harvested at MoD (5 h illumination) using plants hydroponically‐grown to growth stage 5.1 (Boyes et al., 2001). Starch and nitrate were spectrophotometrically analyzed in ethanol extracts through coupled enzymatic assays according to standard protocols (Hendriks et al., 2003; Cross et al., 2006). To quantify absolute ammonium contents, we used the colorimetric method described by Bräutigam et al. (2007).

### 2.6 Statistical analysis

All measurements were performed with 5–8 biological replicates. Statistical differences were determined by two‐tailed Student's *t‐*test (Microsoft Excel 10.0). We used the term significant here if the change in question has been confirmed to be significant at the level of *p < 0.05. The Metaboanalyst 6.0 platform (Ewald et al., 2024) was used to analyze the metabolic data. To visualize the overall changes among the different genotypes and to reduce the dimensionality of the data set, we performed partial least square‐discriminant analysis (PLS‐DA).

## 3 RESULTS

Before undertaking metabolome analyses, we verified that light‐induced, photosynthetically active cell‐specific overexpression of mitochondrial lipoamide dehydrogenase (*mtLPD1*) (for scheme of the transformation construct, see Figure 1A) stimulated GDC activity and photosynthesis‐photorespiratory gas exchange parameters in normal air. In agreement with our earlier report (Timm et al., 2015), we confirmed that leaf‐specific *mtLPD1* overexpression increased GDC activity in isolated mitochondria and stimulated the photorespiratory flux, as evident from the increased photorespiratory CO_2_ release (Figure 1B). Furthermore, at the normal air CO_2_ concentration of 400 ppm, photosynthetic net CO_2_ uptake (*A*_*N*_) increased in the OE lines and this was accompanied by significant increases in stomatal conductance (*g*_*s*_) and the intracellular CO_2_ concentration (*C*_*i*_) (Figure 1C). However, these stimulations were absent at a higher CO_2_ concentration of 2000 ppm (Figure 1D), suppressing photorespiration, confirming that *mtLPD1* overexpression mainly affected the photorespiratory pathway in normal air.

### 3.1 Optimization of glycine decarboxylase lowers daytime photorespiratory intermediates, primarily glycine, inducing specific increases in darkness

**FIGURE 2:**
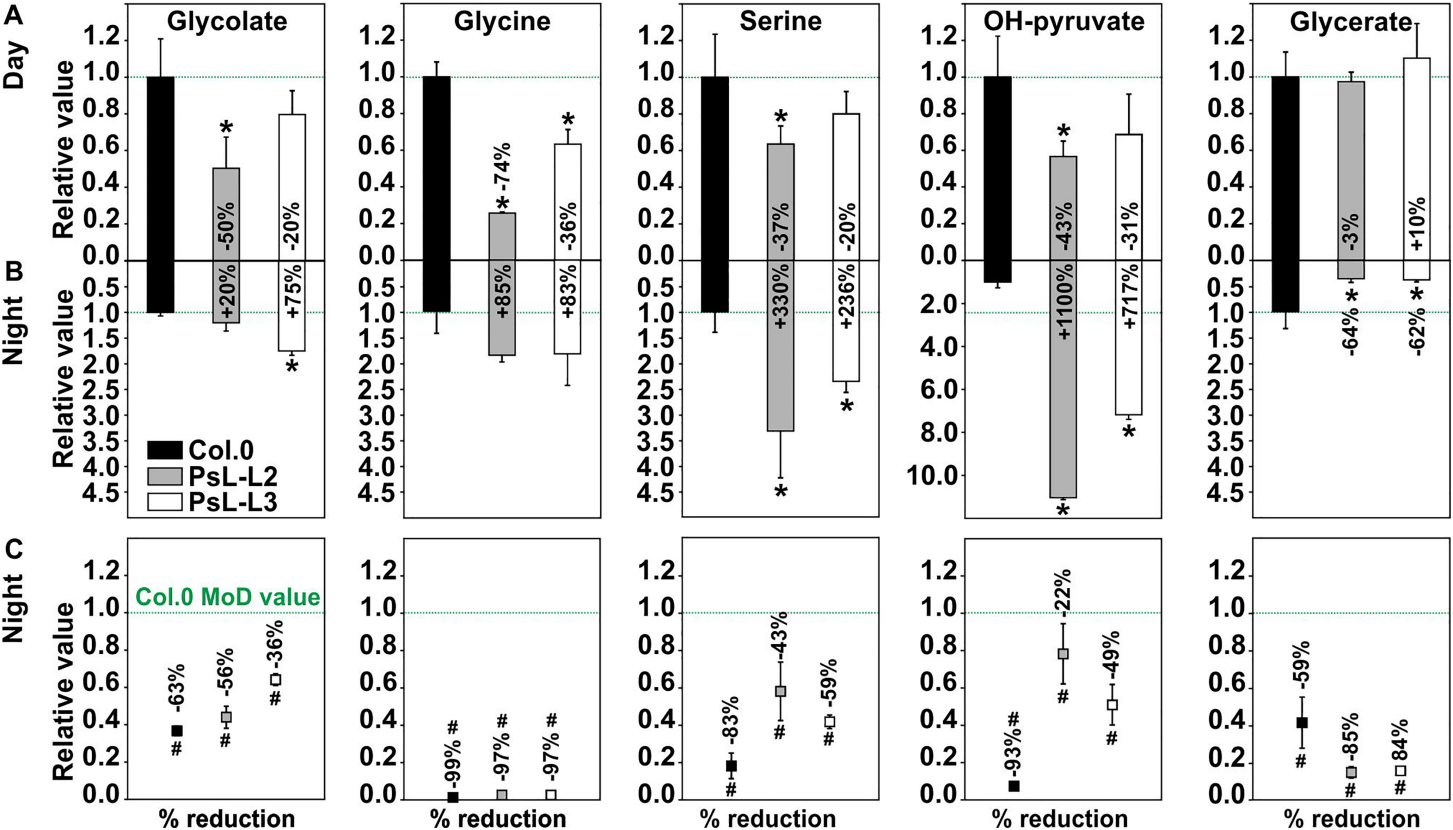
Relative steady‐state abundances of photorespiratory metabolites in leaves of *mtLPD1* overexpressors and the wildtype during illumination and darkness. Shown are five representatives of photorespiratory intermediates in (A) illuminated (MoD, 5 h light) and (B) dark‐adapted leaves (EoD, 13 h darkness) of *mtLPD1‐*overexpressing lines compared to the wildtype. Overexpressor/wildtype ratios of mean steady‐state metabolite contents ± SE (n = 5) are given, with the mean wildtype MoD or EoN values arbitrarily set to 1. (C) Relative steady‐state abundances of photorespiratory intermediates in darkness as percentage of the wildtype MOD value. Asterisks determine values significantly different from the wildtype control at MoD or EoN and rhombs indicate significant changes of relative steady‐state metabolite values measured in the dark (EoN) in comparison to the illuminated (MoD) wildtype based on Student's t‐test (*p < 0.05).

Previous work revealed that leaf‐specific overexpression of *mtLPD1* lowers the steady‐state glycine content in this tissue. This finding, in conjunction with alterations in the ^13^C‐labelling patterns of other photorespiratory intermediates during illumination (Timm et al., 2015) and the increases in GDC activity and photorespiratory CO_2_ release (Figure 1B, C), suggested improved metabolic flux through the photorespiratory cycle. Building on this hypothesis, we extended our analysis to get insight into the steady‐state metabolite accumulation patterns of five selected photorespiratory intermediates (glycolate, glycine, serine, OH‐pyruvate, and glycerate) in both illuminated and dark‐adapted Arabidopsis plants in order to distinguish between conditions with active and inactive photorespiration. In agreement with our earlier study, gas‐chromatography coupled to mass spectrometry (GC–MS) revealed significantly lower glycine accumulation in both transgenic lines (~74% in PsL‐L2; ~36% in PsL‐L3) during the day (Figure 2A). All other photorespiratory intermediates, except for glycerate, also tend to decrease during the day, with significant drops of ~37–50% in our best‐performing line PsL‐L2 (Figure 2A).

In darkness, we observed opposite tendencies. Whilst glycolate (significantly higher in PsL‐L3) and glycine showed minor variations compared to the control, serine (~3.3 fold in PsL‐L2 and ~ 2.4 fold in PsL‐L3) and OH‐pyruvate (~11.0 in PsL‐L2 and ~ 7.2 fold in PsL‐L3) were increased in both *mtLPD1* overexpressors. Otherwise, glycerate was significantly decreased (2.6–2.8‐fold in PsL‐L2 and PsL‐L3) in both transgenics (Figure 2B). However, in comparison with the respective metabolite means of the wildtype measured at MoD, all nighttime relative steady‐state abundances were significantly lower, both in the wildtype and transgenic lines (Figure 2C). Interestingly, the strongest drop in the wildtype was found in glycine (~99%), followed by OH‐pyruvate (~93%), serine (~83%), glycolate (~63%) and glycerate (~59%). Whilst the drop of glycine was similar among the three genotypes, some weaker reductions were seen in glycolate (PsL‐L3), serine (both lines) and OH‐pyruvate (both lines). Again, only glycerate differed from the general trend, with significantly stronger decreased amounts in both overexpression mutants compared to the control (Figure 2B, C). Collectively, our targeted measurements of intermediates involved in photorespiration suggest their faster cycling during illumination and a generally lower abundance at night, where Rubisco‐mediated 2‐PG production and, thus, photorespiration is largely inactive.

### 3.2 Improved leaf‐photorespiration affects the root metabolome

Next, we wanted to address whether optimized photorespiration in leaves has a measurable effect on the metabolism of heterotrophic tissues, especially that of roots. For this purpose, we grew the *mtLPD1‐*overexpressing lines PsL‐L2 and PsL‐L3 alongside the wildtype control hydroponically to facilitate rapid harvesting of leaf and root material from the same plant. GC–MS allowed us to analyze for changes in the relative steady‐state abundances of 50 intermediates particularly related to plant primary carbon and nitrogen metabolism. This dataset was complemented with spectrophotometric measurements of starch, nitrate, and ammonium in the same tissues.

#### 3.2.1 Integrated analysis of metabolite profiles from leaves and roots

**FIGURE 3:**
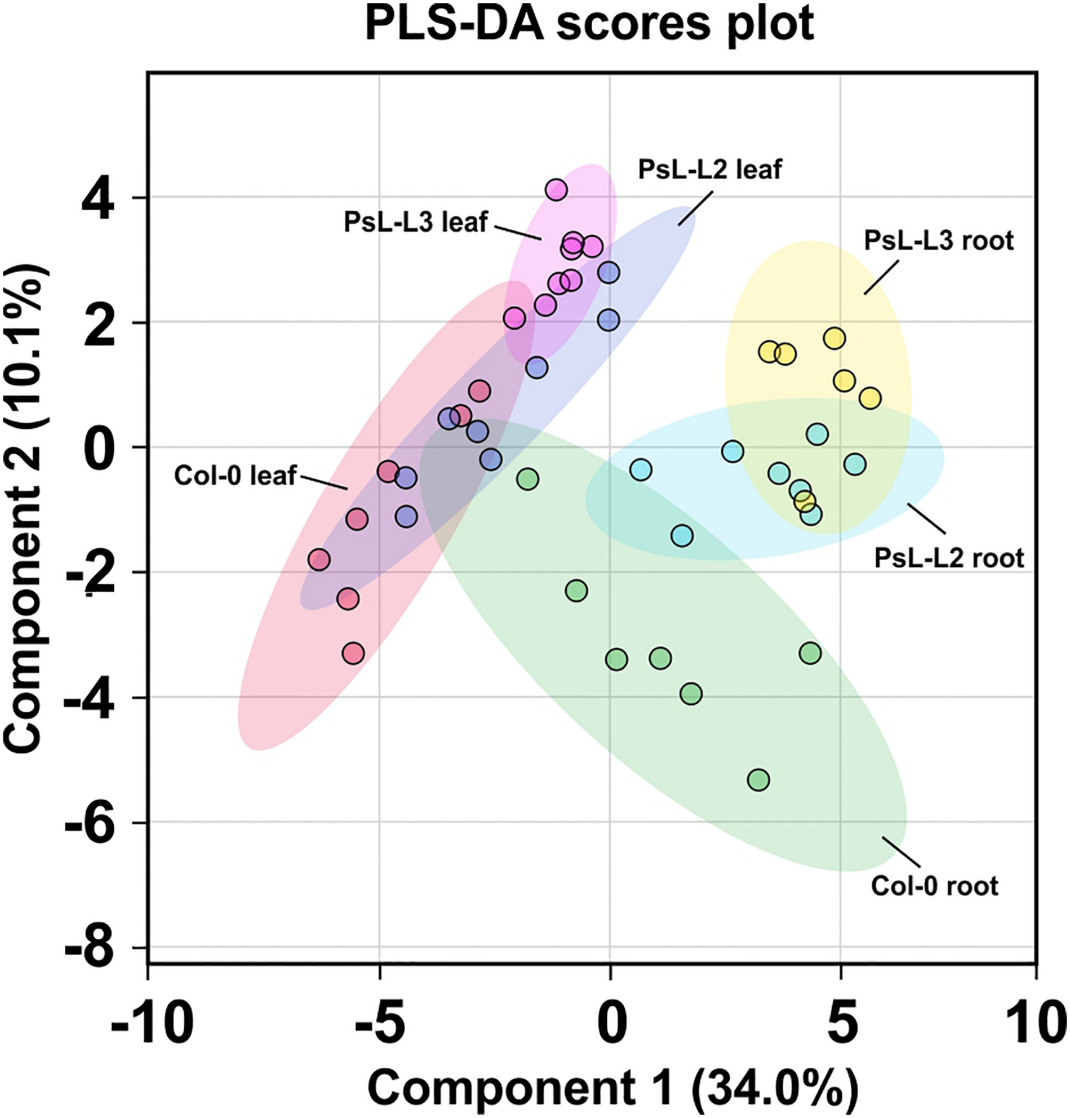
Partial least square‐discriminant analysis (PLS‐DA) of metabolomics data of leaves and roots from the wildtype and *mtLPD1* overexpression plants. (A) PLS‐DA was carried out using metabolomics (GC–MS) data of leaves and roots harvested at mid‐of‐day (MoD) from the wildtype and *mtLPD1* overexpression mutant plants grown hydroponically. The two principal components of the PLS‐DA explain 44.1% of the data. (B) Variable importance in projection (VIP) scores of the PLS‐DA of metabolomics data. The VIP scores, as a representation of the importance of a variable in the PLS‐DA model, of the 25 most representative metabolites are ranked from top to down. Metabolites with values higher than 1.2 indicate metabolites with strong importance for the PLS‐DA model. Colored boxes on the right represent relative concentrations of the metabolites in each genotype and tissue. The analysis was performed using the Metaboanalyst platform 6.0 (Ewald et al., 2024).

To gain a general overview of the metabolic changes in the different genotypes, we first analyzed the full data set using partial least square‐discriminant analysis (PLS‐DA). When leaf and root materials were compared, we observed an almost clear separation of both tissues in the plane of the two first PLS‐DA components, jointly explaining 44.1% of the data variation (Figure 3). More precisely, leaf and root metabolite profiles were mainly separated along component 1 (34%) with high positive loadings (absolute values >0.2) of glutamine, GABA, citrate, tyrosine, glycolate, tryptophan, isoleucine, methionine, maltose, aspartate, malate, succinate and alanine (Table S1). Component 2 was responsible for 10.1% of the variation, mainly comprising high positive loadings of arginine and serine (Figure 3; Table S1). Interestingly, whilst the leaf‐metabolomes of all three genotypes are partially overlapping, the root metabolomes of both transgenic lines shared only a very minor overlap with that of the wildtype and they were separated primarily along component 2 (Figure 3).

#### 3.2.2 Photorespiratory intermediates show different patterns in leaves and roots

**FIGURE 4:**
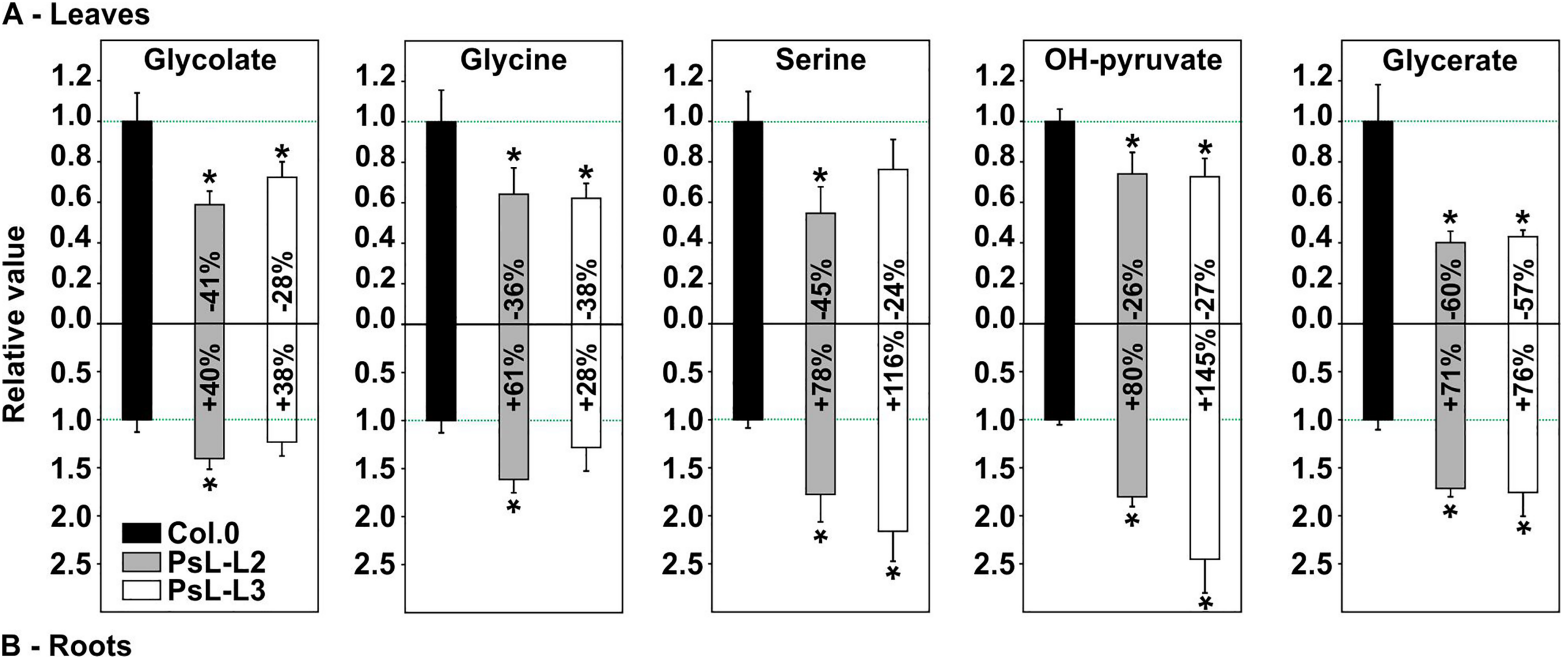
Changes in the relative steady‐state contents of photorespiratory metabolites in leaves and roots of *mtLPD1* overexpressors compared to the wildtype. Five representatives of photorespiratory intermediates are given for (A) leaf tissue and (B) root tissue. Overexpressor/wildtype ratios of mean steady‐state metabolite contents ± SE (n = 5) are shown, where the mean wildtype values are arbitrarily set to 1. Asterisks determine values significantly different from the wildtype control based on Student's t‐test (*p < 0.05).

A targeted view of the dataset, primarily on mtLPD1‐dependent pathways, verified overexpression of *mtLPD1* improved the photorespiratory flux, as expected (Figure 1B). Consequently, the relative steady‐state contents of all five measured representatives of photorespiration (glycolate, glycine, serine, OH‐pyruvate and glycerate) were present at significantly lower abundances in both transgenic lines compared to the wildtype (Figure 4A, except for serine in PsL‐L3). Interestingly, the opposite trend emerged in roots harvested from the same plants. Compared to the wildtype, all five pathway intermediates (except glycolate and glycine in PsL‐L3) were significantly increased in roots of both transgenic lines, with the highest relative increases in serine (78–116%) and OH‐pyruvate (80–145%), followed by glycerate (71–76%) (Figure 4B).

**FIGURE 5:**
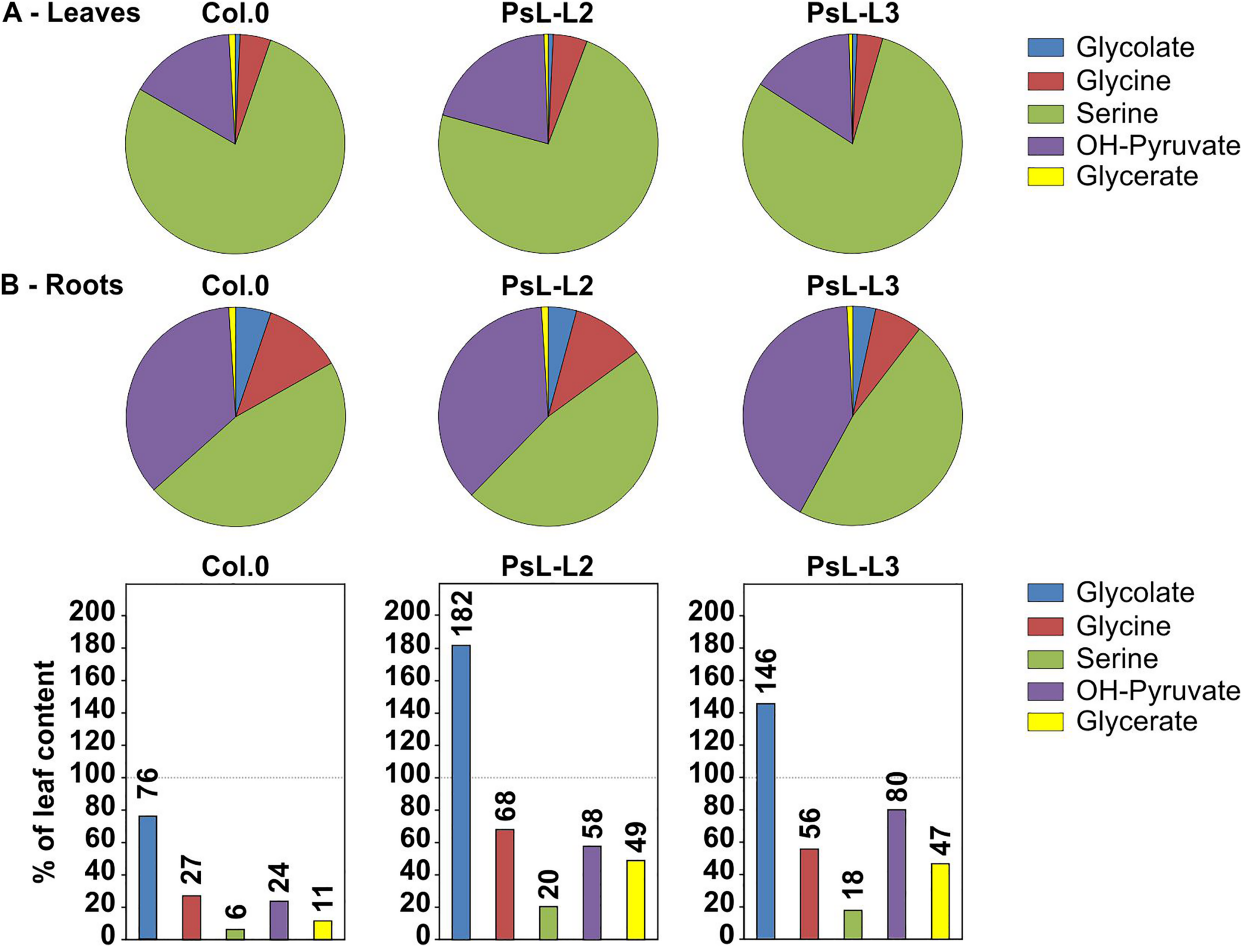
Overview of photorespiratory metabolite compositions in leaves and roots of *mtLPD1* overexpressors compared to the wildtype. Shown are the distributions of photorespiratory intermediates measured as absolute concentrations (nmol * g FW^−1^) in (A) leaves and (B) roots of the wildtype in comparison to the *mtLPD1‐*overexpressing mutants. The lower bar‐chart panel displays the calculated root metabolite amounts compared to the wildtype leaf level in percent. Absolute numerical concentrations of the intermediates are shown in Table 1.

Alongside these observations, we also wanted to obtain insight into the actual pool sizes of the photorespiratory intermediates and quantified them as absolute amounts (Table 1) through GC–MS in both tissues. A general comparison of the five different intermediates revealed serine and OH‐pyruvate accounted for the largest fractions in both leaves and roots (Figure 5A, B). They were followed by glycine, glycolate and glycerate in a descending order. The general pattern of the five intermediates relative to each other was comparable but expectedly varied among the three genotypes in absolute terms according to the afore‐described changes in the relative steady‐state contents (Table 1; Figures 4A, B and 5A, B). When comparing leaf and root tissue, glycolate was the only pathway intermediate present at similar absolute amounts in both tissues in the wildtype and the transgenic lines, again, with the afore‐mentioned slight variations. Further, overexpression of *mtLDP1* led to reduced glycolate in leaves, whilst it accumulated in roots to about 46–82% compared to the control (Table 1; Figure 5B). Apart from glycolate, the wildtype displayed a marked decrease in the absolute contents of all other four photorespiratory intermediates in the roots compared to leaves. More precisely, whilst root glycine and OH‐pyruvate levels dropped to about 27% and 24% of the leaf contents, glycerate and serine showed stronger reductions to 11% and 6%, respectively (Figure 5B; Table 1). Next to these changes, both transgenic lines showed less pronounced reductions in all four photorespiratory intermediates as follows: glycine 56–68%, OH‐pyruvate 58–80%, serine 18–20% and glycerate 47–49% (Table 1; Figure 5B).

**TABLE 1:** Absolute concentrations of photorespiratory intermediates in leaves and roots of hydroponically‐grown wildtype and *mtLPD1‐*overexpressing plants.

nmol * g FW ^*−1*^	*Leaves*		
Intermediate	Col.0	PsL‐L2	PsL‐L3
Glycolate	2.27 ± 0.32	(↓) 1.33 ± 0.15	(↓) 1.64 ± 0.17
Glycine	14.39 ± 2.25	(↓) 9.24 ± 1.88	(↓) 8.95 ± 1.07
Serine	248.37 ± 36.68	(↓) 135.57 ± 32.44	189.22 ± 36.84
OH‐pyruvate	50.03 ± 3.01	(↓) 37.04 ± 5.31	(↓) 36.33 ± 4.53
Glycerate	3.01 ± 0.55	(↓) 1.21 ± 0.17	(↓) 1.30 ± 0.10
nmol * g FW ^*−1*^	*Roots*		
Intermediate	Col.0	PsL‐L2	PsL‐L3
Glycolate	1.73 ± 0.22	(↑) 2.43 ± 0.19*	(↑) 2.39 ± 0.26*
Glycine	3.89 ± 0.50*	(↑) 6.28 ± 0.54	(↑) 4.98 ± 0.96*
Serine	15.53 ± 1.33*	(↑) 27.58 ± 4.47*	(↑) 33.53 ± 4.88*
OH‐pyruvate	11.86 ± 0.63*	(↑) 21.37 ± 1.22*	(↑) 29.07 ± 6.53
Glycerate	0.34 ± 0.04*	(↑) 0.59 ± 0.03*	(↑) 0.60 ± 0.09*

Plants were grown hydroponically under environmentally controlled conditions to growth stage 5.1 (Boyes et al., 2001). Leaf and root tissues were harvested from the same plant at MoD and subjected to GC–MS analysis. Values are means ± SE (*n* = 5) and are given in nmol * g FW^−1^. Values in bold letters indicate significant changes of the transgenic lines compared to the wildtype (*p < 0.05) and asterisks indicate significant changes of the root compared to the leaf of each genotype.

#### 3.2.3 The TCA‐cycle intermediates largely mirror the photorespiratory pattern

**FIGURE 6:**
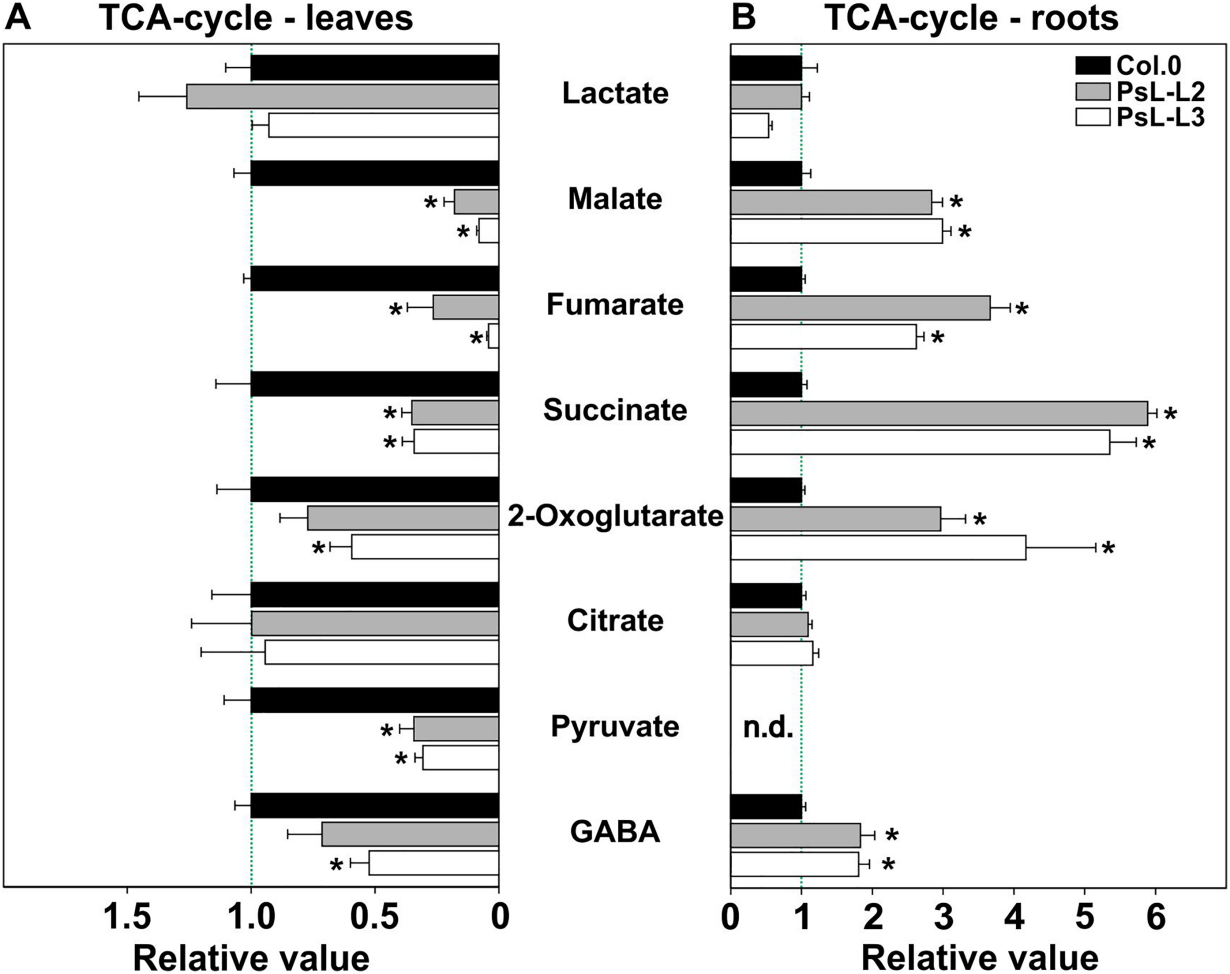
Changes in steady‐state contents of TCA‐cycle and related metabolites in leaves and roots of *mtLPD1* overexpressors compared to the wildtype. Shown are eight TCA‐cycle and associated metabolites in (A) leaf tissue and (B) root tissue. Overexpressor/wildtype ratios of mean steady‐state metabolite contents ± SE (n = 5) are shown, where the mean wildtype values are arbitrarily set to 1. Asterisks determine values significantly different from the wildtype control based on Student's t‐test (*p < 0.05). n.d. – not determined.

Given the TCA‐cycle closely interacts with photorespiration (Obata et al., 2016) and relies on two mtLPD1‐dependent multienzymes, namely pyruvate dehydrogenase complex (PDC) and 2‐oxoglutarate dehydrogenase complex (OGDC), we also inspected the relative steady‐state contents of several TCA‐cycle and associated intermediates in more detail. Most of the analyzed metabolites (malate, fumarate, succinate, 2‐oxoglutarate [only in PsL‐L3], pyruvate and GABA [only in PsL‐L3]) were significantly less abundant in leaves of both transgenic lines compared to wildtype, whereas lactate and citrate values were similar among all analyzed genotypes (Figure 6A). On the other hand, we found significantly increased amounts of malate, fumarate, succinate, 2‐oxoglutarate and GABA in roots of the transgenic lines in comparison to the control. Again, lactate and citrate were invariant among all analyzed genotypes, whilst pyruvate was below the detection limit (Figure 6B).

#### 3.2.4 Amino acid metabolism differentially responds to mtLPD1 overexpression

Apart from the photorespiratory amino acids glycine and serine, we also compared the accumulation patterns in leaves and roots of 17 other amino acids (16 proteinogenic and 1 non‐proteinogenic) among the studied genotypes. In leaves, we found three groups of amino acids sharing a consistent pattern, with the first being increased (arginine, asparagine and methionine), a second with decreased steady‐state contents (alanine, aspartate, glutamate, OH‐proline, proline), and a third with mostly unchanged content (glutamine, isoleucine, lysine, phenylalanine, threonine, tyrosine and valine) in the transgenic lines compared to the control (Table S2). In roots, only two groups of consistently responding amino acids were seen: one with a generally increased cotent between transgenic and wildtype (alanine, aspartate, glutamate, glutamine, phenylalanine, tryptophan and tyrosine) and the second with mostly unaltered values (arginine, asparagine, isoleucine, methionine) (Table S2).

#### 3.2.5 Soluble sugars, starch and nitrogen compounds

**FIGURE 7:**
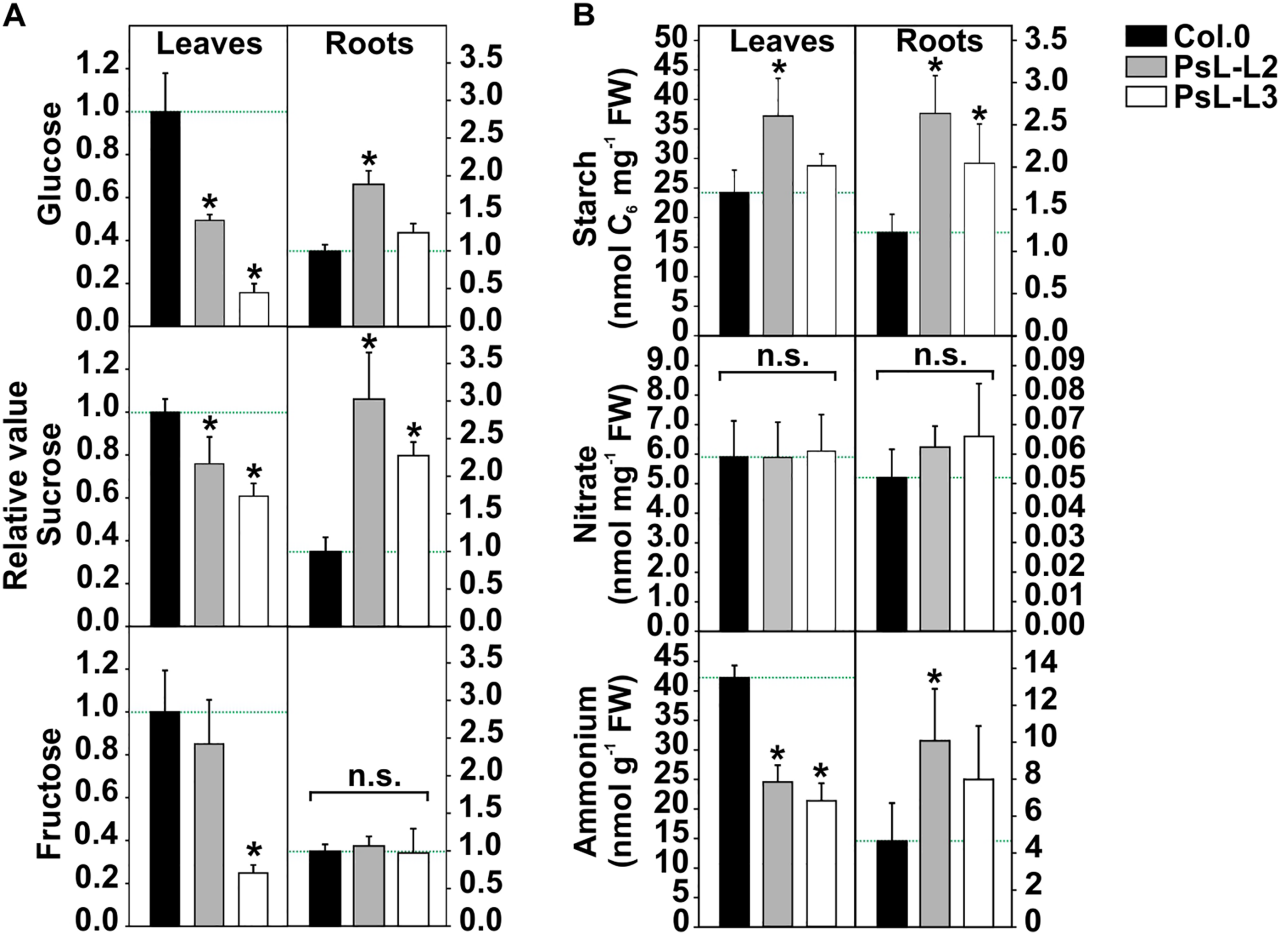
Selected carbohydrates, nitrate and ammonium in leaves and roots of *mtLPD1* overexpressors compared to the wildtype. Plants were grown hydroponically under environmentally controlled conditions to growth stage 5.1 (Boyes et al., 2001). Leaf and root tissues were harvested from the same plant at mid‐of‐day (MoD) and used for GC–MS or enzymatic analysis. (A) Relative steady‐state contents of selected soluble sugars (glucose, sucrose and fructose). Overexpressor/wildtype ratios of mean steady‐state metabolite contents ± SE (n = 5) are shown, where the mean wildtype leaf‐ or root‐values are arbitrarily set to 1. (B) Absolute contents (mean ± SD; *N* = 5) of starch, nitrate and ammonium in the wildtype and *mtLPD1‐*overexpressing lines. Asterisks determine values significantly different from the wildtype control based on Student's t‐test (*p < 0.05). n.s. – not significant.

Another interesting observation was made when looking at the soluble sugars. Most of the analyzed representatives showed a general decrease in the leaves of the transgenic lines, including glucose, sucrose, fructose (only PsL‐L3), erythritol, galactinol, isomaltose, maltose, raffinose (only PsL‐L3), trehalose, and xylose (Figure 7A; Table S2). On the other hand, several sugars tended to accumulate in the roots of the transgenic lines, especially glucose (significant only in PsL‐L2), sucrose (2–3 fold compared to the control) and erythritol (significant only in PsL‐L2). Apart from this response, a broad range of other sugars were rather unchanged (galactinol, raffinose, trehalose and xylose) or below the detection limit (isomaltose, maltose and xylose) in these tissues (Figure 7A; Table S3).

The main storage carbohydrate, starch, behaved slightly differently from the soluble sugars. Hence, starch was increased in leaves (only significant in PsL‐L2) and roots of the transgenic lines compared to the control (Figure 7A). In light of the changes in carbohydrate metabolism, 2‐oxoglutarate, glutamate and glutamine, we also analyzed the absolute amounts of nitrate and ammonium among the genotypes. Whilst the first was found equally distributed among all genotypes in source and sink tissue, ammonium mirrored the photorespiratory pattern: i.e. lower in leaves and higher in roots of the transgenic lines in comparison to the wildtype (Figure 7B).

#### 3.2.6 Responses of other intermediates of central metabolism

Among 10 other components related to central plant metabolism, we found a consistent pattern in ascorbate and dehydroascorbate (DH‐ascorbate), which were both lower in the leaves of the transgenic lines compared to the control. DH‐ascorbate was increased around 2.5‐to‐3‐fold in the roots of the transgenics, whilst ascorbate was below detection limit (Tables S2 and 3). Similar trends were seen in guanidine, inositol and threonate, which were all lower in transgenic leaves, and ethanolamine and guanidine, both significantly lower in roots (Tables S2 and 3).

### 3.3 Alterations in the metabolic pattern of phloem exudates

**FIGURE 8:**
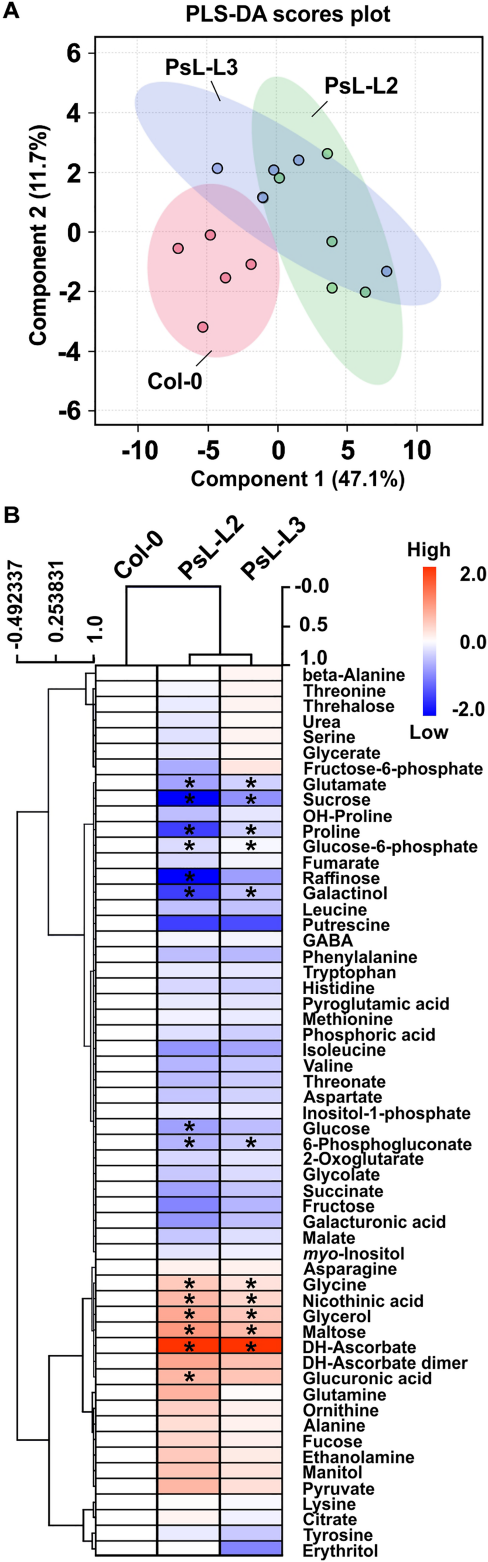
PLS‐DA analysis and heatmap of relative steady‐state metabolite contents in phloem exudates of the wildtype and the *mtLPD1* overexpression plants at MoD. Plants were grown on soil under environmentally controlled conditions to growth stage 5.1 (Boyes et al., 2001). Leaves were harvested at MoD to collect phloem exudates, which were subsequently subjected to GC–MS analysis. (A) PLS‐DA of the metabolomics (GC–MS) data of phloem exudates. The two principal components of the PLS‐DA explain 58.8% of the data. The analysis was performed using the Metaboanalyst platform 6.0 (Ewald et al., 2024). (B) Heatmap of the full metabolite dataset. Displayed are mean steady‐state metabolite contents (*n* > 4; log2 of x‐fold). Metabolites marked with an asterisk are significantly different from the control (p < 0.05).

Our results showed a considerable effect on the root metabolome in response to leaf‐specific overexpression of *mtLPD1*. Therefore, we next asked wether the phloem metabolite compositions differ among the transgenic lines and the wildtype, eventually explaining the observed metabolic shifts between leaves and roots. For this reason, we collected phloem exudates at MoD and subjected them to GC–MS‐based metabolite analysis. We identified 57 metabolites in phloem extracts. PLS‐DA of the dataset revealed a clear separation of both transgenic lines from the wildtype along the first two PLS‐DA components, jointly explaining 58.8% of the data variation (Figure 8A). Separation along principal component 1 (47.1%) had high positive loadings (absolute values >0.2) of glycine, fucose, maltose, glucuronic acid, nicotinic acid, and tryptophan. Component 2 was responsible for 11.7% of data variation, mainly driven by high positive loadings of the three photorespiratory metabolites glycerate, glycolate, and serine, as well as trehalose, urea, 2‐oxoglutarate, glucose‐6‐phosphate, fructose‐6‐phosphate, succinate, glutamate, phosphoric acid and inositol‐1‐phosphate (Table S4).

Further elaboration of the dataset (for heatmap overview, see Figure 8B; numerical values are given in Table S5) revealed that only a few metabolites were significantly altered in response to *mtLPD1* overexpression. On the one hand, five compounds were consistently increased in both lines, namely DH‐ascorbate (20.7 and 8.8‐fold), glycine (2.2 and 1.9‐fold), maltose (2.0 and 1.6‐fold), glycerol (1.8 and 1.4‐fold), and nicotinic acid (1.6 and 1.3‐fold). Glucuronic acid also tended to increase (1.6‐fold), but only significantly in PsL‐L2 (Figure 8B). On the other hand, consistent decreases were observed for glutamate (both ~0.6‐fold), proline (0.2 and 0.4‐fold), glucose‐6‐phosphate (both 0.2‐fold), galactinol (0.4 and 0.5‐fold), and 6‐phosphogluconate (both 0.3‐fold). Further changes appeared in sucrose (0.2 and 0.4‐fold), raffinose (0.2 and 0.6‐fold) and glucose (0.5 and 0.6‐fold), which were only significant in PsL‐L2 (Figure 8B; Table S5). In summary, changes in the phloem exudate composition were mostly restricted to carbohydrate, amino acid and redox‐metabolism.

## 4 DISCUSSION

Photorespiration has been identified as one of the main processes limiting carbon assimilation in crop plants. Therefore, many attempts have been undertaken to tackle this problem (e.g. Kebeish et al., 2007; Betti et al., 2016; Eisenhut et al., 2019; South et al., 2019). Previously, we showed that optimization of endogenous photorespiration through overexpression of specific components, such as *GDC* or *PGLP*, stimulates photosynthesis and plant growth (Timm et al., 2012; 2015; Simkin et al., 2017; Flügel et al., 2017). However, it was yet unknown if light‐induced, leaf‐specific engineering of photorespiration (Figure 1A) has any effect on the metabolism of non‐photosynthesizing tissue or sink organs, including that of roots. Alongside our metabolome analyses, we verified that light‐induced, leaf‐specific overexpression of *mtLPD1* impacted GDC activity and the photorespiratory flux. Similar to the initial, comprehensive description of the transgenic lines (Timm et al., 2015), we confirmed upregulated GDC activity in isolated mitochondria, ultimately leading to increased photorespiratory CO_2_ release, as measured through gas exchange analysis (Figure 1B). Hence, these findings supported our hypothesis of increased flux through the photorespiratory pathway. Furthermore, a higher photorespiratory flux (i.e. improved turnover of critical intermediates) was assumed to be causative for stimulated photosynthesis in normal air due to alleviated negative feedback on the Calvin‐Benson cycle (Fernie and Bauwe, 2020). Indeed, and as reported previously, we measured higher photosynthetic CO_2_ assimilation alongside increased stomatal conductance and the internal CO_2_ concentration at the air CO_2_ level (400 ppm) (Figure 1C). The changes were, however, negligible when CO_2_ was increased to 2000 ppm. Statistically, non‐significant changes in photosynthetic parameters under conditions with suppressed photorespiration support our conclusion that overexpression of *mtLPD1* mostly affects GDC and photorespiration. This statement agrees with our previous report on unpromoted growth of *mtLPD1* overexpression mutants grown in high CO_2_ (Timm et al., 2015), and further suggested only minor effects on photosynthesis and growth due to alterations in other mtLPD1‐dependent pathways, including the TCA‐cycle and the degradation of branched‐chain amino acids.

### 4.1 Improved GDC impacts not only daytime but also nighttime steady‐state pools of photorespiratory intermediates

In agreement with previous findings on GDC manipulations (Timm et al., 2012; 2015), we measured significant reductions in the steady‐state contents of photorespiratory intermediates in illuminated leaves during this study, suggesting their accelerated turnover (Figure 2A). Interestingly, light‐induced, leaf‐specific optimization of GDC activity through *mtLPD1* overexpression (Figure 1B) also caused yet unreported, specific metabolic changes in photorespiratory intermediates in darkness (Figure 2B). In the wildtype, photorespiratory intermediate levels are much lower in darkness than during daytime (Figure 2C), which was consistent with inactive RuBP oxygenation by Rubisco in darkness and, thus, negligible 2‐PG formation (Ogren et al., 1986; Orr et al., 2023). The very strong reductions in the wildtype steady‐state pools of glycine (99%), serine (83%) and OH‐pyruvate (93%) suggested that these intermediates are rather exclusively formed and cycled through active photorespiration. This view is supported by a recent labeling study showing that especially glycine and serine are actively exported from the photorespiratory cycle to fuel other cellular sinks instead of only being used to refeed the C3 cycle (Fu et al., 2023). Moreover, carbon and nitrogen sequestered in glycine and serine are also used for protein or glutathione biosynthesis, sulfur metabolism and nitrogen assimilation (Noctor et al., 1999; Ros et al., 2014; Abadie and Tcherkez, 2019; Fu et al., 2023). The relatively moderate drops in glycolate and glycerate (~40%; Figure 2B, C) might imply that neither are exported or share only limited overlap with other metabolic branches during darkness. This hypothesis finds support by the relatively small absolute steady‐state concentrations of both metabolites in leaves compared to serine and OH‐pyruvate (Table 1; Figure 4A). Nevertheless, rather minor variations in leaf and root glycolate amounts were also seen in other studies, including those featuring enzymes participating in glycolate metabolization (Nunes‐Nesi et al., 2014; Kerchev et al., 2016), suggesting a relatively stable steady‐state pool compared to other pathway intermediates. In contrast, glycerate is actively transported to the vacuole under certain conditions (Lin and Tsay, 2023; Timm and Eisenhut, 2023), eventually preventing the visibility of smaller fluctuations of the steady‐state pool.

Interestingly, *mtLPD1* overexpression lines showed significant deviations from the wildtype light–dark pattern (Figure 2A, B). A specific pattern emerged for photorespiratory intermediates downstream of the GDC reaction, namely, serine, OH‐pyruvate and glycerate. Whilst glycerate was the only photorespiratory intermediate significantly reduced in both transgenic lines, OH‐pyruvate and serine clearly accumulated during darkness in both transgenic lines (Figure 2B). It seems reasonable to assume that the faster turnover of photorespiratory intermediates during the day in the overexpressor lines, especially that of glycine and serine, caused the internal pool sizes to fall below a critical threshold. In order to prevent the cell from potential glycine or serine starvation, the phospho‐serine pathway starting at 3‐PGA can be activated by replenishing serine and also glycine via serine‐hydroxymethyltransferase (Ros et al., 2014; Kleczkowski and Igamberdiev, 2024). This supports experimental evidence results from a study on serine:glyoxylate aminotransferase (SGAT) overexpression plants, which counteract the strong decrease in the daytime serine steady‐state content through induction of the phosphorylated pathway of serine biosynthesis (Modde et al., 2017). Future enhancements of the photorespiratory cycle, thus, should consider potential trade‐offs due to depleted pathway intermediates.

### 4.2 Optimized photorespiration in leaves impacts the metabolite composition of heterotrophic roots

Despite a comparably good understanding of the metabolic consequences of optimized photorespiration in photosynthesizing source leaves, there was a clear lack of knowledge wether these changes are actually communicated onto sink tissue, especially roots. Therefore, we performed a metabolome analysis of leaves and roots of hydroponically‐grown *mtLPD1‐*overexpressing lines alongside the wildtype. The general overview of metabolic responses obtained through PLS‐DA revealed similar patterns in leaves, whilst an almost complete separation of the wildtype and both transgenic lines was seen for roots (Figure 3A). From this result, we conclude that, indeed, engineering of leaf‐photorespiration feeds back onto heterotrophic sink tissue. Among the compounds driving the separation, we found a clear enrichment of metabolites involved in photorespiration and other mtLPD1‐dependent and ‐associated pathways, supporting the causality of the genetic manipulation and its consequences.

When looking on the absolute and relative steady‐state values of photorespiration intermediates and the steady‐state TCA‐cycle and associated metabolite abundances in more detail, we found an almost consistent pattern. In leaves, all five photorespiratory and most TCA‐cycle intermediates are significantly reduced in the overexpressor lines compared to the control (Table 1; Figures 4A and 6A). Moreover, the results are supportive of a stimulated flux through both pathways in response to *mtLPD1* overexpression. Notably, roots appear to respond exactly the other way around. Almost all representatives of both pathways are significantly increased in roots. Given that mtLPD1 levels are similar between roots of the wildtype and the transgenic lines and overexpression is restricted to leaves (Timm et al., 2015), these changes cannot be directly explained through altered mtLPD1‐dependent enzyme activities. One possible explanation would be an increased transport of photorespiratory intermediates from leaves to roots through the petiole, presumably as glycine. Whilst some experimental evidence is in favor of the hypothesis (Madore and Grodzinski, 1984), a recent modelling study concluded this fraction might be comparably small (Fu et al., 2023). However, further targeted research would be required to fully resolve the actual fractions of photorespiratory intermediates to be transported from source to sink tissues, especially in light of the altered absolute metabolite concentrations in roots of the transgenic lines compared to the wildtype (Figure 5A, B). With regards to the increased TCA‐cycle and other glycolytic/respiratory intermediates, it seems reasonable to speculate that optimized leaf‐photorespiration and the resulting stimulation of photosynthesis and growth potentially signals back onto the root tissue to induce respiratory metabolism for balancing metabolism. Mechanistically, higher TCA‐cycle activity is likely needed to support the root tissue's higher demand on the reducing equivalents NADH and FADH_2_ to drive the mitochondrial electron transport chain (e.g. Martínez‐Reyes and Chandel, 2020). Interestingly, a recent study comparing developing Arabidopsis and maize roots suggested that some of the TCA‐cycle intermediates are involved in the control of root development (Zhang et al., 2023). Since we observed a high accumulation of succinate and 2‐oxoglutarate in the two *mtLPD1* overexpressors (Figure 6A, B), one could conclude that increased rosette biomass in these plants signals the need to simultaneously increase root tissue to maintain efficient nutrient uptake. Therefore, a detailed characterization of root growth, architecture and metabolism with different nutrient supplementations is required to shed more light on this hypothesis.

Beyond the metabolic changes observed for photorespiration and the TCA‐cycle, which are two mtLDP1‐depending metabolic pathways, other factors driving metabolite cluster separation included soluble sugars, sugar derivates and some amino acids (Figure 3A, B; Table S1). Although the impact of all compounds is not always obvious, it is likely to assume that the increased availability of photosynthates due to higher photosynthetic rates in *mtLPD1* overexpressors (Figure 1B) results in specific metabolic shifts beyond the ones in participating pathways. Hence, the surplus of carbon can be used to either increase the biosynthesis of storage carbohydrates [i.e. transitory starch in leaves and roots (Figure 7A, B), higher accumulation and transport of soluble sugars] or the production of amino acids to support higher protein synthesis (Figure 7A, B; Tables S2, S3). Interestingly, the observed shifts in carbohydrates also seem to affect nitrogen metabolism, as high N‐containing amino acids were observed to accumulate. Ammonia, however, displayed the same accumulation pattern as observed for photorespiratory intermediates (Figure 7B; Tables S2, S3). More comprehensive studies are needed to elucidate the interplay of increased photorespiratory fluxes and nitrogen assimilation and metabolism.

### 4.3 The metabolite profiles of phloem exudates differ between the wildtype and the mtLPD1 overexpressors

In light of the changes in root metabolism following leaf‐specific *mtLPD1* overexpression, changes in metabolite transport from photosynthesizing source organs into heterotrophic sink tissue could potentially explain the observed changes. Indeed, PLS‐DA revealed an almost clear separation of phloem exudates between wildtype and the two transgenic lines (Figure 8A). This result provides a hint that *mtLPD1* overexpression could specifically impact phloem exudates. The changes involved the key photorespiratory intermediate glycine, which was also increased in the root metabolome (Figure 4B). Given both *mtLPD1* overexpressors consistently shared significantly increased abundances of glycine in their phloem exudates, we favor this intermediate as the major export metabolite from photorespiring leaves to roots. Recent ^13^C‐labelling and modelling studies support the unique role of glycine, especially during photorespiratory transients (Fu et al., 2022). The authors hypothesize that glycine can accumulate in large quantities as it is less critical to metabolism than other photorespiratory intermediates and, thus, could support photosynthesis under fluctuating environments. This statement and the fact that glycine can be used to transport redox‐equivalents between tissue (Timm and Hagemann, 2020) support the idea of glycine being transported through the phloem to root tissue, where it can be stored on a longer‐term (Figure 4B). However, not all effects are specifically restricted to photorespiration, given there were also high impacts associated with sugars, TCA cycle intermediates or amino acids (Figure 8A, B; Tables S4, S5). These findings clearly imply that at least some of the changes in the root metabolome can be well related to increased export of these compounds from the accumulated pools in the source into the sink organs. Future studies using mutants in specific carbohydrate or amino acids transporters are required to fully elucidate the underlying mechanisms.

## 5 CONCLUSION

Our comprehensive metabolic study demonstrated that light‐induced, photosynthetically active cell‐specific optimization of photorespiration (Figure 1A, B) has not only consequences for illuminated, photosynthesizing (source) tissue, but is also translated onto other plant tissues, including dark‐adapted leaves and heterotrophic (sink) root tissue. Specific increases in soluble sugars and transitory starch stocks in roots of the transgenic lines suggest a higher export of the carbon surplus fixed in transgenic leaves (Figure 1C) to this storage organ. Consequently, this strategy has the potential to increase the yield of beet‐ or tuber‐forming plants such as sugar beet, potato or cassava.

## AUTHOR CONTRIBUTIONS

S.T., A.R.F., and H.B. designed the research. S.T., A.F., S.A., and K.J. performed the research. S.T., A.F., and S.A. analyzed the data. A.R.F. and M.H. provided the metabolite analysis platform and experimental tools. S.T. wrote the article with revisions received from M.H., and A.R.F.

## Supporting information


**Supplemental Table S1.** Loadings of different metabolites on the first two principal components (PCs) in leaf‐ and root‐tissue harvested from the wildtype and the *mtLPD1* overexpressors.
**Supplemental Table S2**. Relative steady‐state metabolite contents in leaves of the wildtype in comparison with the *mtLPD1* overexpressors at MoD.
**Supplemental Table S3**. Relative steady‐state metabolite contents in roots of the wildtype in comparison with the *mtLPD1* overexpressors at MoD.
**Supplemental Table S4**. Loadings of different metabolites on the first two principal components (PCs) in phloem exudates obtained from the wildtype and the *mtLPD1* overexpressors.
**Supplemental Table S5**. Relative steady‐state metabolite contents in phloem exudates of the wildtype in comparison with the *mtLPD1* overexpressors at MoD.

## Data Availability

The data that support the findings of this study are available from the corresponding author upon reasonable request.
