# Supplementary material for: Improved photorespiration has a major impact on the root metabolome of Arabidopsis
Source: Physiol Plant. 2025 Mar 3;177(2):e70142. doi: 10.1111/ppl.70142 (PMC11876089; doi:10.1111/ppl.70142)
Supplement: Supplementary file 1 — Supplemental Table S1. Loadings of different metabolites on the first two principal components (PCs) in leaf‐ and root‐tissue harvested from the wildtype and the mtLPD1 overexpressors. Supplemental Table S2. Relative steady‐state metabolite contents in leaves of the wildtype in comparison with the mtLPD1 overexpressors at MoD. Supplemental Table S3. Relative steady‐state metabolite contents in roots of the wildtype in comparison with the mtLPD1 overexpressors at MoD. Supplemental Table S4. Loadings of different metabolites on the first two principal components (PCs) in phloem exudates obtained from the wildtype and the mtLPD1 overexpressors. Supplemental Table S5. Relative steady‐state metabolite contents in phloem exudates of the wildtype in comparison with the mtLPD1 overexpressors at MoD. [file PPL-177-e70142-s001.pdf]

Supplemental Data

Improved photorespiration has a major impact on the root metabolome of Arabidopsis

Stefan Timm[1*], Alexandra Florian[2], Saleh Alseekh[2], Kathrin Jahnke[1], Martin Hagemann[1], Alisdair R. Fernie[2], Hermann Bauwe[1]

[1]University of Rostock, Plant Physiology Department, Albert-Einstein-Straße 3, D-18059 Rostock, Germany

[2]Max Planck Institute of Molecular Plant Physiology, Am Mühlenberg 1, D-14476 Golm, Germany

***Correspondence to:**

Stefan Timm,

Email stefan.timm@uni-rostock.de

Supplemental Table S1. Loadings of different metabolites on the first two principal components (PCs) in leaf- and root-tissue harvested from the wild type and the *mtLPD1* overexpressors.

Summary of the numerical values of the loadings on the first two PCs of the PLS-DA shown in Figure 2 are given. Loadings with the absolute values >0.2 (strong impact) are shown in bold.

Metabolite	PC1	PC2
Glutamine	**0.25897**	-0.13527
GABA	**0.23887**	-0.19241
Citrate	**0.23775**	**-0.21739**
Tyrosine	**0.23533**	-0.06677
Valine	**0.23419**	-0.10008
Glycolate	**0.23337**	-0.16477
Tryptophan	**0.23087**	-0.08615
Isoleucine	**0.22365**	-0.12675
Methionine	**0.22301**	-0.19570
Maltose	**0.22161**	**-0.24915**
Aspartate	**0.21943**	-0.11998
Malate	**0.21720**	-0.13621
Succinate	**0.21565**	-0.05263
Alanine	**0.21335**	-0.12954
Phenylalanine	0.19587	0.03884
Lactate	0.18668	**-0.20038**
Xylose	0.18609	**-0.30093**
OH-Pyruvate	0.18587	0.02027
beta-Alanine	0.18416	0.08891
Glycerol	0.18127	-0.17376
Ethanolamine	0.17934	-0.07538
Glycine	0.17902	-0.12637
Glutamate	0.17240	-0.09091
2-Oxoglutarate	0.16555	-0.01561
Lysine	0.15774	**-0.21907**
Sucrose	0.14847	-0.10167
Threonine	0.11589	0.12954
Trehalose	0.09423	-0.31545

Ammonium	0.07415	0.01170
Arginine	0.04595	**0.21274**
Asparagine	0.04055	0.15605
Starch	0.03293	0.11302
Glycerate	0.01322	-0.14988
4-hydroxyproline	0.00486	-0.09973
Putrescine	-0.01346	0.09160
Dehydroascorbate	-0.04688	-0.10462
Serine	-0.04870	**0.21276**
Threonate	-0.05048	-0.10431
Nitrate	-0.05709	0.14077
Guanidine	-0.06558	-0.00486
Erythritol	-0.07382	0.18009
Glucose	-0.07654	-0.06911
Raffinose	-0.10776	-0.08813
Fructose	-0.12140	-0.05950
Fumarate	-0.13737	**-0.21877**
Ascorbate	-0.13761	0.16542
Proline	-0.14754	0.13225
Inositol	-0.16590	-0.04156
Galactinol	-0.19959	-0.11237
Isomaltose	**-0.20524**	-0.04921
Pyruvate	**-0.22384**	0.10016

Supplemental Table S2. Relative steady-state metabolite contents in leaves of the wild type in comparison with the *mtLPD1* overexpressors at MoD.

Plants were grown hydroponically under environmental controlled conditions to growth stage 5.1 (Boyes et al., 2001). Leaf- and root-tissue was harvested from the same plant at MoD and subjected to GC-MS analysis. Overexpressor/wild-type ratios of mean steady-state metabolite contents ± SE (n = 5) are shown, where the mean wild-type leaf-values are arbitrarily set to 1. Bold letters indicate values that are significantly different from the wild-type control based on Student's t test (*$p < 0.05$). n.d. – not detectable.

Intermediate	***Leaves (relative values) – mid of day***		
	**Col.0**	**PsL-L2**	**PsL-L3**
Amino acids			
Alanine	1.00 ± 0.088	**0.53 ± 0.045**	**0.59 ± 0.100**
ß-Alanine	1.00 ± 0.156	1.22 ± 0.175	**1.55 ± 0.161**
Arginine	1.00 ± 0.642	**3.79 ± 1.045**	**5.37 ± 0.779**
Asparagine	1.00 ± 0.090	**3.22 ± 1.113**	**4.16 ± 0.904**
Aspartate	1.00 ± 0.058	**0.55 ± 0.109**	**0.36 ± 0.048**
Glutamate	1.00 ± 0.029	**0.73 ± 0.061**	**0.80 ± 0.032**
Glutamine	1.00 ± 0.085	0.95 ± 0.109	1.21 ± 0.075
Isoleucine	1.00 ± 0.084	1.78 ± 0.390	1.09 ± 0.177
Lysine	1.00 ± 0.083	1.82 ± 0.491	2.98 ± 1.027
Methionine	1.00 ± 0.165	**1.95 ± 0.489**	**1.54 ± 0.140**
OH-Proline	1.00 ± 0.065	**0.48 ± 0.076**	**0.51 ± 0.037**
Phenylalanine	1.00 ± 0.061	0.91 ± 0.091	1.33 ± 0.234
Proline	1.00 ± 0.049	**0.44 ± 0.115**	**0.60 ± 0.069**
Threonine	1.00 ± 0.078	1.28 ± 0.141	1.25 ± 0.167
Tryptophan	1.00 ± 0.109	1.07 ± 0.262	**0.59 ± 0.032**
Tyrosine	1.00 ± 0.058	1.87 ± 0.458	1.08 ± 0.082
Valine	1.00 ± 0.092	1.18 ± 0.160	1.30 ± 0.294
Sugars			
Erythritol	1.00 ± 0.056	**0.55 ± 0.100**	**0.56 ± 0.058**
Galactinol	1.00 ± 0.290	**0.27 ± 0.063**	**0.02 ± 0.005**

Isomaltose	1.00 ± 0.280	**0.38 ± 0.051**	**0.03 ± 0.005**
Maltose	1.00 ± 0.265	**0.53 ± 0.078**	**0.38 ± 0.109**
Raffinose	1.00 ± 0.041	0.89 ± 0.071	**0.18 ± 0.042**
Threhalose	1.00 ± 0.216	**0.45 ± 0.077**	**0.23 ± 0.059**
Xylose	1.00 ± 0.133	**0.63 ± 0.086**	**0.40 ± 0.041**
Other metabolites			
Ascorbate	1.00 ± 0.349	**0.23 ± 0.056**	0.57 ± 0.195
Dehydroascorbate	1.00 ± 0.071	**0.52 ± 0.096**	**0.58 ± 0.076**
Ethanolamine	1.00 ± 0.162	1.82 ± 0.356	0.84 ± 0.082
Gluconate-6-phosphate	1.00 ± 0.066	0.62 ± 0.283	**0.52 ± 0.064**
Glycerol	1.00 ± 0.036	**0.55 ± 0.100**	1.05 ± 0.024
Guanidine	1.00 ± 0.131	**0.50 ± 0.064**	**0.31 ± 0.029**
Inositol	1.00 ± 0.067	**0.44 ± 0.136**	**0.23 ± 0.014**
Putrescine	1.00 ± 0.394	1.89 ± 0.223	1.38 ± 0.027
Threonate	1.00 ± 0.073	**0.52 ± 0.060**	**0.44 ± 0.038**

Supplemental Table S3. Relative steady-state metabolite contents in roots of the wild type in comparison with the *mtLPD1* overexpressors plants at MoD.

Plants were grown hydroponically under environmental controlled conditions to growth stage 5.1 (Boyes et al., 2001). Leaf- and root-tissue was harvested from the same plant at MoD and subjected to GC-MS analysis. Overexpressor/wild-type ratios of mean steady-state metabolite contents ± SE (n = 5) are shown, where the mean wild-type root-values are arbitrarily set to 1. Bold letters indicate values that are significantly different from the wild-type control based on Student's t test (*$p < 0.05$). n.d. – not detectable.

Intermediate	***Roots (relative values) – mid of day***		
	**Col.0**	**PsL-L2**	**PsL-L3**
Amino acids			
Alanine	1.00 ± 0.109	**2.27 ± 0.257**	**1.47 ± 0.173**
ß-Alanine	1.00 ± 0.201	1.39 ± 0.087	**2.88 ± 0.83**
Arginine	1.00 ± 0.052	0.93 ± 0.098	0.80 ± 0.162
Asparagine	1.00 ± 0.120	1.18 ± 0.208	1.54 ± 0.43
Aspartate	1.00 ± 0.072	**1.39 ± 0.078**	**2.03 ± 0.138**
Glutamate	1.00 ± 0.111	**1.29 ± 0.056**	**1.84 ± 0.189**
Glutamine	1.00 ± 0.084	**1.45 ± 0.064**	**1.44 ± 0.055**
Isoleucine	1.00 ± 0.030	1.55 ± 0.301	1.30 ± 0.270
Lysine	1.00 ± 0.071	1.15 ± 0.132	**0.26 ± 0.034**
Methionine	1.00 ± 0.087	1.15 ± 0.142	1.05 ± 0.314
OH-Proline	1.00 ± 0.122	**1.55 ± 0.108**	1.10 ± 0.045
Phenylalanine	1.00 ± 0.047	**3.22 ± 0.466**	**3.23 ± 0.333**
Proline	1.00 ± 0.089	**1.55 ± 0.141**	1.03 ± 0.170
Threonine	1.00 ± 0.062	**1.73 ± 0.223**	1.66 ± 0.354
Tryptophan	1.00 ± 0.058	**2.10 ± 0.388**	**2.44 ± 0.653**
Tyrosine	1.00 ± 0.100	**2.44 ± 0.557**	**2.78 ± 0.513**
Valine	1.00 ± 0.040	**1.76 ± 0.342**	1.63 ± 0.344
Sugars			
Erythritol	1.00 ± 0.214	**2.08 ± 0.365**	1.24 ± 0.015
Galactinol	1.00 ± 0.646	1.73 ± 0.239	1.27 ± 0.944

Isomaltose	n.d.	n.d.	n.d.
Maltose	n.d.	n.d.	n.d.
Raffinose	1.00 ± 0.054	1.53 ± 0.375	1.89 ± 1.090
Threhalose	1.00 ± 0.086	1.23 ± 0.124	1.13 ± 0.272
Xylose	1.00 ± 0.254	0.64 ± 0.081	n.d.
Other metabolites			
Ascorbate	n.d.	n.d.	n.d.
Dehydroascorbate	1.00 ± 0.028	**1.68 ± 0.168**	**2.13 ± 0.220**
Ethanolamine	1.00 ± 0.062	**2.52 ± 0.135**	**3.03 ± 0.700**
Gluconate-6-phosphate	1.00 ± 0.114	1.04 ± 0.127	1.16 ± 0.175
Glycerol	1.00 ± 0.030	0.95 ± 0.054	0.98 ± 0.049
Guanidine	1.00 ± 0.062	**1.99 ± 0.143**	**2.83 ± 0.711**
Inositol	1.00 ± 0.142	**2.56 ± 0.457**	0.62 ± 0.115
Putrescine	1.00 ± 0.072	**1.73 ± 0.224**	0.70 ± 0.056
Threonate	1.00 ± 0.224	1.00 ± 0.109	0.85 ± 0.120

Supplemental Table S4. Loadings of different metabolites on the first two principal components (PCs) in phloem exudates obtained from the wild type and the *mtLPD1* overexpressors.

Summary of the numerical values of the loadings on the first two PCs of the PLS-DA shown in Figure 7 are given. Loadings with the absolute values >0.2 (strong impact) are shown in bold.

Metabolite	PC1	PC2
Glycine	**0.2132**	0.0242
Fucose	**0.2076**	-0.1364
Maltose	**0.2061**	-0.1131
Glucuronic acid	**0.2044**	-0.1340
Nicotinic acid	**0.2034**	-0.0025
Tryptophan	**0.2019**	-0.1628
beta-Alanine	0.1990	0.0628
Lysine	0.1983	-0.1878
Glycerol	0.1982	-0.1604
GABA	0.1941	-0.1819
Pyruvate	0.1935	-0.1380
Tyrosine	0.1933	**-0.2305**
myo-Insitol	0.1919	-0.1067
Ethanolamine	0.1914	-0.1949
Sucrose	-0.1908	0.1228
Alanine	0.1895	-0.0039
Raffinose	-0.1865	-0.0013
Dehydroascorbate dimer	0.1836	-0.1423
Manitol	0.1790	**-0.2053**
Dehydroascorbate	0.1734	**-0.2498**
Leucine	0.1691	**-0.2099**
Threonine	0.1688	0.0949
Asparagine	0.1665	0.0229
Phenylalanine	0.1659	**-0.2339**
Histidine	0.1601	**-0.2124**
Methionine	0.1575	-0.1233
Threhalose	0.1571	**0.2475**
Urea	0.1509	**0.2712**
2-Oxoglutarate	0.1467	**0.2178**

Glycerate	0.1458	**0.2879**
Aspartate	0.1455	-0.0235
Serine	0.1451	**0.2145**
Pyroglutamic acid	0.1395	0.0279
Galactoinol	-0.1339	0.0601
Fumarate	0.1326	0.0118
Citrate	0.1214	-0.0341
Glutamine	0.1173	-0.0013
Proline	-0.1172	0.1015
Valin	0.1124	-0.0784
Isoleucine	0.1116	-0.1806
Malate	0.0985	0.0155
Fructose-6-phosphate	-0.0842	**0.2366**
Threonate	0.0797	0.0802
Putrescine	-0.0770	-0.1201
Galacturonic acid	0.0757	-0.0185
Glucose	0.0728	**-0.2895**
Glucose-6-phosphate	-0.0557	**0.3354**
Succinate	0.0530	**0.2449**
Erythritol	0.0445	0.0525
Fructose-6-phosphate	0.0419	**-0.3344**
Glutamate	-0.0362	**0.3146**
Phosphoric acid	-0.0313	**0.2382**
Glycolate	0.0249	**0.3469**
Inositol-1-phosphate	0.0113	**0.2919**

Supplemental Table S5. Relative steady-state metabolite contents in phloem exudates of the wild type in comparison with the *mtLPD1* overexpressors at MoD.

Plants were grown on soil under environmental controlled conditions to growth stage 5.1 (Boyes et al., 2001). Leaves were harvested at MoD to collect phloem exudates which were subsequently subjected to GC-MS analysis. Displayed are mean steady-state metabolite contents ± SE (n = 5). Metabolites marked in bold are significantly different from the control based on Student's t test (p < 0.05).

Intermediate	***Phloem exudates (relative values) – mid of day***		
	**Col.0**	**PsL-L2**	**PsL-L3**
Alanine	165.00 ± 32.26	206.73 ± 13.01	181.20 ± 22.85
ß-Alanine	0.25 ± 0.07	0.25 ± 0.03	0.27 ± 0.03
Asparagine	7.92 ± 1.59	8.70 ± 1.20	8.68 ± 1.55
GABA	31.06 ± 6.09	29.46 ± 2.75	29.37 ± 3.01
Glutamine	0.19 ± 0.05	0.32 ± 0.05	0.19 ± 0.05
Glycine	3.69 ± 0.91	**8.17 ± 0.67**	**6.88 ± 0.66**
Histidine	1.31 ± 0.40	1.06 ± 0.13	1.01 ± 0.15
Isoleucine	16.56 ± 6.47	9.34 ± 1.05	10.14 ± 0.86
Leucine	20.42 ± 7.05	14.62 ± 1.63	14.66 ± 1.38
Lysine	3.55 ± 0.85	3.62 ± 0.38	3.44 ± 0.32
Methionine	0.97 ± 0.12	0.91 ± 0.11	0.87 ± 0.13
Ornithine	0.14 ± 0.03	0.19 ± 0.01	0.16 ± 0.02
Phenylalanine	3.72 ± 1.10	2.61 ± 0.22	2.53 ± 0.24
Proline	128.16 ± 32.70	**26.40 ± 5.45**	**48.83 ± 13.81**
Putrescine	0.11 ± 0.06	0.04 ± 0.00	0.04 ± 0.00
Serine	68.74 ± 17.71	58.15 ± 5.80	74.23 ± 11.10
Threonine	23.37 ± 5.99	22.29 ± 2.53	25.08 ± 4.55
Tryptophan	1.46 ± 0.33	1.30 ± 0.12	1.29 ± 0.14
Tyrosine	0.20 ± 0.05	0.18 ± 0.02	0.15 ± 0.02
Valin	41.51 ± 12.85	27.90 ± 3.57	30.75 ± 5.01
Aspartate	119.97 ± 14.63	87.72 ± 8.84	93.95 ± 8.46

Citrate	106.35 ± 13.97	114.31 ± 10.97	98.96 ± 17.26
DH-Ascorbate	0.02 ± 0.01	**0.35 ± 0.10**	**0.12 ± 0.09**
Fumarate	208.20 ± 40.87	169.58 ± 14.34	196.26 ± 26.95
Glutamate	90.19 ± 10.98	**54.93 ± 10.45**	**51.93 ± 13.17**
Glycerate	1.84 ± 0.46	1.62 ± 0.27	1.94 ± 0.20
Glycolate	0.35 ± 0.09	0.26 ± 0.06	0.29 ± 0.07
Malate	68.39 ± 14.16	50.49 ± 4.85	57.35 ± 9.76
Nicotinic acid	0.50 ± 0.08	**0.81 ± 0.08**	**0.66 ± 0.06**
Pyroglutamic acid	128.97 ± 21.70	115.33 ± 14.34	111.94 ± 20.08
Succinate	8.90 ± 2.90	5.35 ± 0.94	6.47 ± 0.99
Threonate	1.93 ± 0.21	1.33 ± 0.18	1.46 ± 0.15
Fructose	641.62 ± 154.68	330.85 ± 35.45	431.34 ± 53.48
Fucose	2.39 ± 0.62	3.15 ± 0.38	2.65 ± 0.31
Glucose	95.68 ± 24.14	**49.12 ± 4.52**	57.46 ± 6.56
Maltose	0.11 ± 0.02	**0.22 ± 0.02**	**0.18 ± 0.02**
Raffinose	2.53 ± 0.40	**0.51 ± 0.16**	1.49 ± 0.45
Sucrose	1359.16 ± 358.70	**332.89 ± 93.36**	**474.89 ± 148.62**
Threhalose	0.34 ± 0.08	0.30 ± 0.05	0.36 ± 0.04
Gluconate-6-phosphate	0.08 ± 0.02	**0.03 ± 0.01**	**0.03 ± 0.01**
Glucose-6-phosphate	0.39 ± 0.09	**0.08 ± 0.02**	**0.07 ± 0.03**
Glycerol	19.53 ± 3.21	**35.22 ± 5.28**	**28.01 ± 5.36**
Inositol-1-phosphate	0.18 ± 0.03	0.16 ± 0.04	0.16 ± 0.05
Manitol	13.52 ± 4.69	20.07 ± 3.34	16.24 ± 2.16
Phosphoric acid	168.51 ± 17.49	142.34 ± 22.78	129.34 ± 30.11
OH-Proline	0.12 ± 0.01	0.08 ± 0.01	0.10 ± 0.01
Urea	5.58 ± 1.31	4.90 ± 0.77	5.81 ± 1.47
DH-Ascorbate dimer	0.13 ± 0.05	0.24 ± 0.04	0.20 ± 0.08
Ethanolamine	10.94 ± 3.13	15.72 ± 2.45	12.60 ± 2.81
Fructose-6-phosphate	0.06 ± 0.02	0.04 ± 0.02	0.08 ± 0.07
Galactoinol	10.73 ± 1.22	**3.77 ± 0.85**	**5.10 ± 1.47**
Galacturonic acid	0.66 ± 0.26	0.37 ± 0.06	0.47 ± 0.08

Glucuronic acid	0.12 ± 0.02	**0.20 ± 0.03**	0.18 ± 0.02
2-Oxoglutarate	2.16 ± 0.53	1.74 ± 0.23	1.87 ± 0.21
myo-Insitol	32.98 ± 4.69	28.56 ± 1.64	30.30 ± 1.66
Pyruvate	4.02 ± 1.00	6.57 ± 0.84	5.01 ± 0.71
Erythritol	0.11 ± 0.03	0.11 ± 0.04	0.05 ± 0.00
